# Scleral remodeling in early adulthood: the role of FGF-2

**DOI:** 10.1038/s41598-023-48264-5

**Published:** 2023-11-27

**Authors:** Yingyan Qin, Taixiang Liu, Zhaotian Zhang, Shuwen Xing, Li Gong, Yao Ni

**Affiliations:** 1 State Key Laboratory of Ophthalmology, Zhongshan Ophthalmic Center, Sun Yat-Sen University, Guangdong Provincial Key Laboratory of Ophthalmology and Visual Science, 54S Xianlie Road, Guangzhou, 510060 China; 2 Guizhou Ophthalmic Hospital, The Affiliated Hospital of Zunyi Medical University, Zunyi, 563003 China; 3 Instrumental Analysis and Research Center, Sun Yat-Sen University, 135W Xingang Road, Guangzhou, 510275 China

**Keywords:** Extracellular signalling molecules, Growth factor signalling, Ageing, Transdifferentiation

## Abstract

Emmetropization, a natural process of ocular elongation, is closely associated with scleral remodeling. The Fibroblast growth factor-2 (FGF-2) was reported involved in scleral remodeling in myopia models. Herein, we aimed to investigate the role of scleral fibroblast-to-myofibroblast differentiation and FGF-2 in scleral remodeling during maturation. Our findings revealed that the posterior scleral fibroblasts (SFs) from mature guinea pigs exhibit increased stiffness compared to those from young guinea pigs. Moreover, mature SFs displayed decreased cell proliferation but increased levels of α-SMA, matrix metalloproteinase 2 (MMP2), and collagen 1, when compared to young SFs. Additionally, the mRNA expression of scleral *Fgf-2*, *Fgf receptor 1 (Fgfr1)*, *Fgfr2*, *Fgfr3*, and *Fgfr4* was increased in mature SFs. Notably, exogenous FGF-2 showed increased cell proliferation and led to decreased expression of α-SMA, MMP2, and collagen 1 in mature SFs. Overall, our findings highlight the influence of maturation on SFs from posterior scleral shells, resulting in increased stiffness and the manifestation of fibroblast-to-myofibroblast differentiation during development. Exogenous FGF-2 increased cell proliferation and reversed the age-related fibroblast-to-myofibroblast differentiation, suggesting a potential role of FGF-2 in regulating scleral remodeling.

## Introduction

The natural process whereby the eye moves toward an ideal refractive state, referred to as emmetropization, depends largely on matching eye length to the focal length of the eye^1,2^. Results from clinical and experimental studies clearly demonstrate an emmetropization mechanism that functions to decrease refractive error by means of modifications in scleral remodeling during the postnatal progression of the eye^3,4^. In human populations, emmetropization frequently becomes disturbed, possibly as a result of the influences exerted by the environment and/or genetics, thereby result in the development of myopia^4^. An understanding of the mechanism underlying emmetropization is necessary for the development of successful therapeutic strategies to prevent myopia.

Ocular elongation is a result of extracellular matrix (ECM) remodeling that takes place in the scleral shell. Numerous studies have shown a decelerated synthesis of scleral ECM, along with an accelerated degradation in the axial myopia^5^. Sclera is known to be a dynamic tissue that undergoes constant remodeling throughout life^3^. The sclera is mainly composed of collagen, proteoglycans, glycoproteins, and other ECM components, scleral fibroblasts (SFs) play a crucial role in regulating tissue strength and resilience in the sclera, due to their production of postnatal ECM molecules^3^. Scleral myofibroblasts, contractile cells, are another scleral cell type that is differentiated from SFs. ECM remodeling in the sclera can trigger fibroblast-to-myofibroblast differentiation^6^. Studies indicate that fibroblasts transform into myofibroblasts and upregulate α-SMA, which augments their ability to generate contractile force and increase ECM stiffness in myopic eyes^7–9^. Because the sclera undergoes remodeling throughout life, it is possible that the composition of scleral cell types and cell biology change during maturation and as sclera ages. The following characteristics are additionally associated with aged sclera: impaired proliferative capacity, reduced hyaluronan production, maintained or reduced collagen production, and increased tissue density^10–12^.

Fibroblast growth factor-2 (FGF-2) has been shown to be a potent growth factor that regulates SF proliferation^13^. More importantly, FGF-2 involved in sclera remodeling and prevents the development of myopia^14^. The developmental-related changes in scleral remodeling and the possible role of FGF-2 remained unclear. The guinea pig eye presents a valuable model for investigating the regulation of ocular growth due to its similarity to the human eye in terms of axial growth^15,16^. Therefore, our study seeks to investigate the role of scleral fibroblast-to-myofibroblast differentiation and FGF-2 in scleral remodeling during maturation in guinea pig. Understanding the scleral remodeling during emmetropization, a normal ocular elongation process, may help us better understand myopia, an excessive ocular elongation process.

## Results

### Posterior SF stiffness increased with sclera maturation

**Figure 1:**
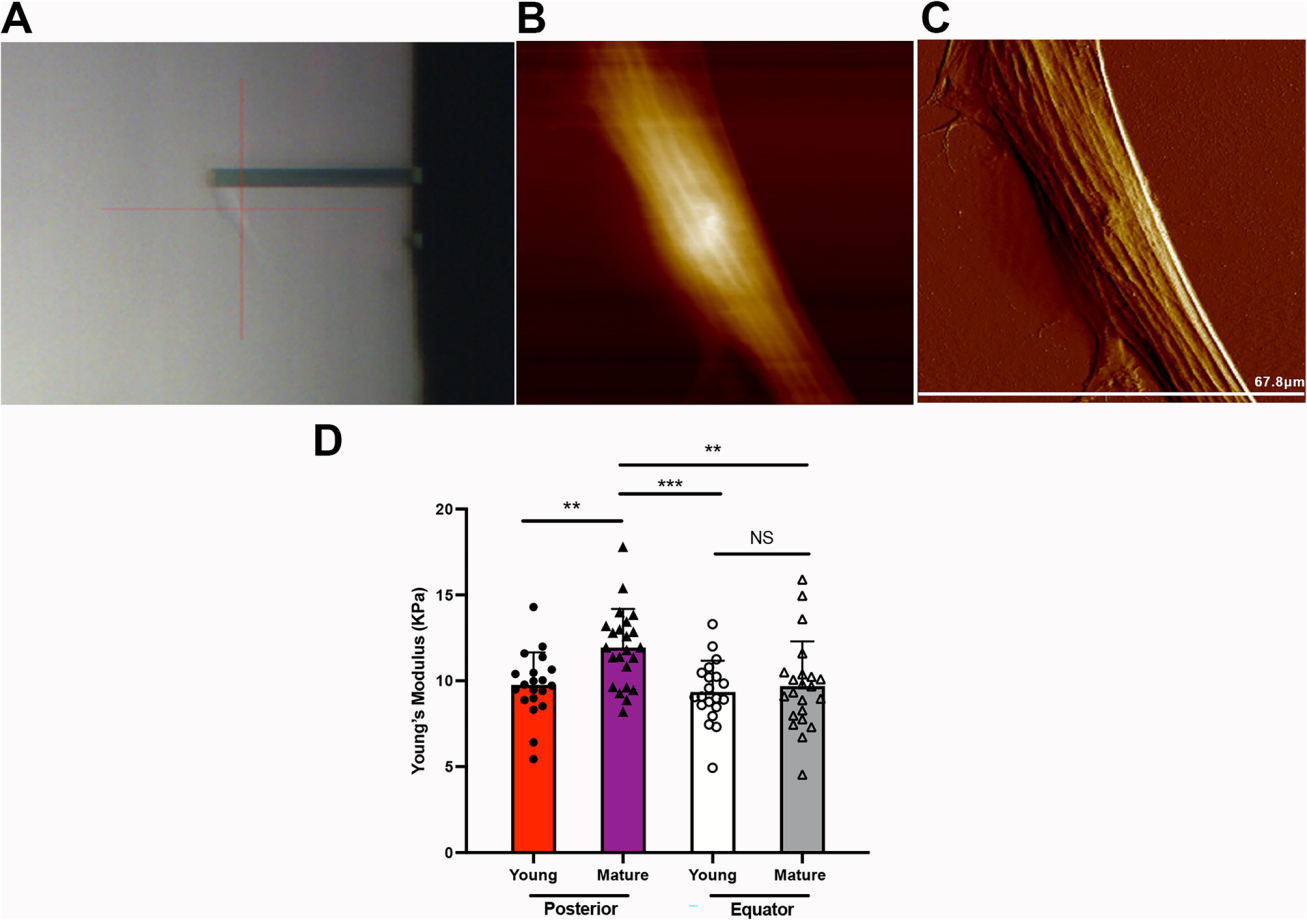
The biomechanical properties of scleral fibroblasts (SFs). (**A**) SFs were scanned using an atomic force microscopy (AFM) probe. A representative 2-D image (**B**) and 3-D image (**C**) of living SFs. (**D**) The Young’s modulus values of SFs from the posterior shell and equator. **P < 0.01, ***P < 0.001, NS: not significant, n = 20.

Previous studies have indicated that age is associated with the stiffness of sclera tissue^11^. To further clarified of changes in the biomechanical characteristic of the sclera, we quantitatively analyzed the biomechanical change and obtained morphological data in the living SFs using atomic force microscopy (AFM) (Fig. 1A–C). The Young’s modulus was significantly higher in the posterior SFs of mature guinea pigs than in those of the young guinea pigs (11.95 ± 2.24 kPa vs. 9.76 ± 1.90 kPa, *P* < 0.01) (Fig. 1D). Meanwhile, the Young’s modulus did not show statistical significance when comparing the equatorial SFs of the mature guinea pigs to those of the young guinea pigs (9.69 ± 2.61 kPa vs. 9.36 ± 1.82 kPa, *P* > 0.05). Interestingly, the mature SFs from posterior scleral shells were stiffer than the mature SFs from equatorial scleral shells, as evidenced in a significantly higher Young’s modulus (11.95 ± 2.24 kPa vs. 9.69 ± 2.61 kPa, P < 0.01). These data demonstrate that the SFs from posterior scleral shells were affected by natural maturation, and the posterior SFs from mature guinea pigs were stiffer than those from young guinea pigs. Given this finding, the following study used only SFs from posterior scleral shells.

### SFs exhibit myofibroblast transition during sclera maturation

**Figure 2:**
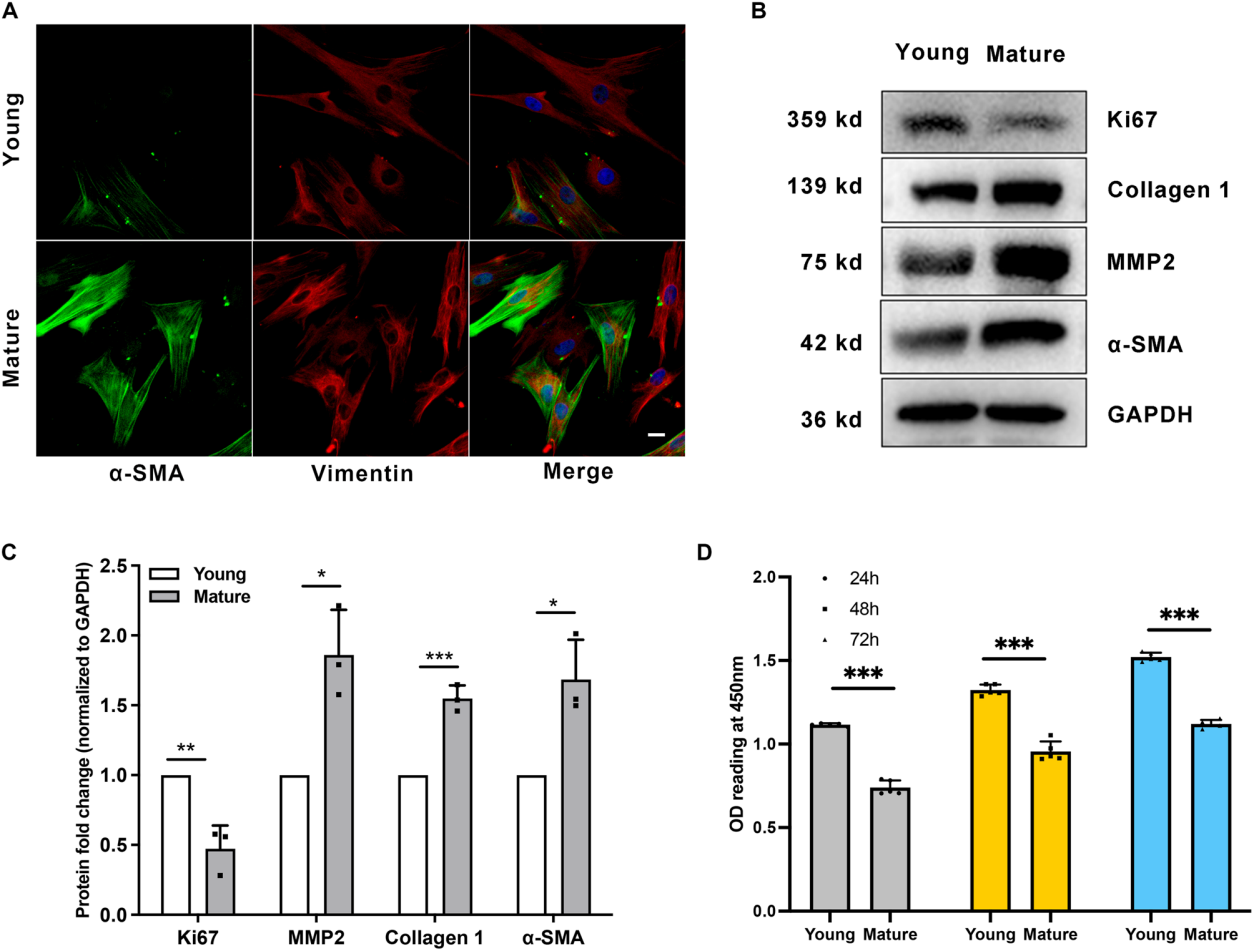
Scleral fibroblasts (SFs) exhibit myofibroblast transition during sclera maturation. SFs from young (four-week-old) and mature (six-month-old) pigmented guinea pigs were used in the study. (**A**) SFs were probed for α-SMA (green), Vimentin (red), and DAPI (blue). Scale bar: 20 μm. (**B**) Western blot analysis was performed to probe for Ki67 (359 kDa), collagen 1 (139 kDa), MMP2 (75 kDa), and α-SMA (42 kDa). GAPDH was used as an internal control. (**C**) Quantification of the protein expression levels in (**B**). The fold change relative to the control group level is displayed. **P < 0.01, *P < 0.05, n = 3. (**D**) Cell proliferation was assessed by CCK-8 assay after culture for 24 h, 48 h, and 72 h. ***P < 0.001, n = 5.

To further determine whether SFs exhibit myofibroblast transition during sclera maturation, we examined the expression of α-SMA and collagen 1^17^, which are markers of myofibroblast and ECM changes. Increased α-SMA expression in mature SFs was demonstrated using immunofluorescence and western blot (Fig. 2A–C). As shown in Fig. 2B, C, MMP2 and collagen 1 increased in mature guinea pigs’ SFs. Cell proliferation marker Ki67 was decreased in mature guinea pigs’ SFs. Consistently, young guinea pigs’ SFs were more proliferative, as the young group had significantly higher OD values than that of the mature group (1.12 ± 0.009 *versus* 0.74 ± 0.042 at 24 h, *P* < 0.001; Fig. 2D). These results indicate that SFs exhibit myofibroblast transition and ECM changes during natural maturation.

### The role of FGF-2 in scleral remodeling

**Figure 3:**
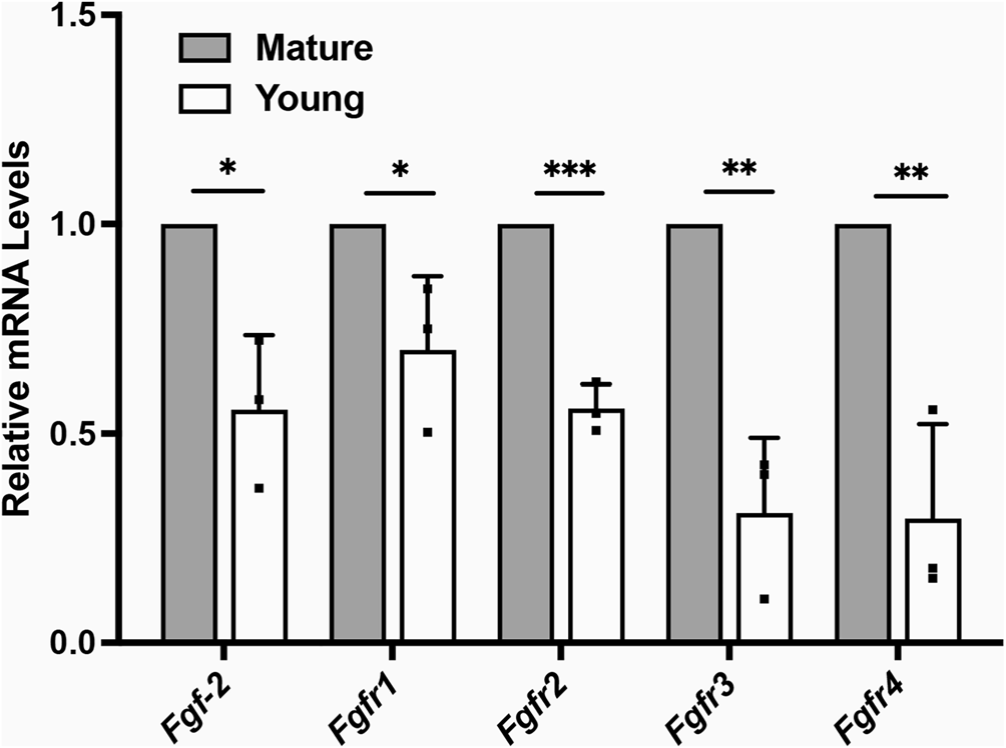
The mRNA expression levels of *Fgf-2* and *Fgfrs* in Scleral fibroblasts (SFs). Total RNA was extracted from young (four-week-old) and mature (six-month-old) SFs. The mRNA level of *Fgf-2*, *Fgfr1*, *Fgfr2*, *Fgfr3*, and *Fgfr4* was determined using real-time PCR and normalized to *GAPDH*. ***P < 0.001, **P < 0.01, *P < 0.05, n = 3.

FGF-2 is an effective mediator of sclera remodeling and ocular elongation^14,18^. First, we assessed the mRNA expression levels of *Fgf*-2 and *Fgf receptors (Fgfrs)* in SFs. The real-time PCR showed that *Fgf-2*, *Fgfr1*, *Fgfr2*, *Fgfr3*, and* Fgfr4* were increased in mature SFs (Fig. 3).

**Figure 4:**
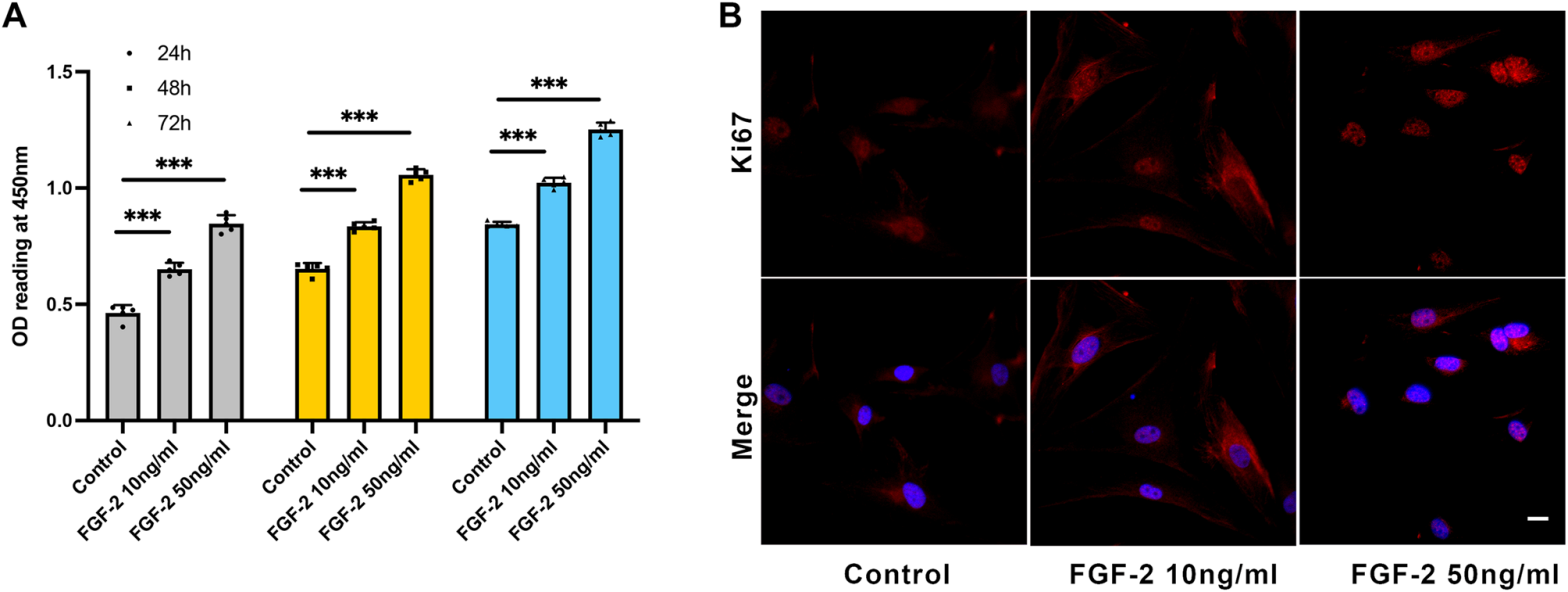
Scleral fibroblast proliferation was improved by FGF-2. (A) Cell proliferation was assessed by CCK-8 assay after 10 ng/ml and 50 ng/ml FGF-2 treatment for 24 h, 48 h, and 72 h. ***P < 0.001, n = 5. (B) Mature scleral fibroblasts were treated either with or without FGF-2 for 24 h. Cells were probed for Ki67 (red) and DAPI (blue). Scale bar: 20 μm.

**Figure 5:**
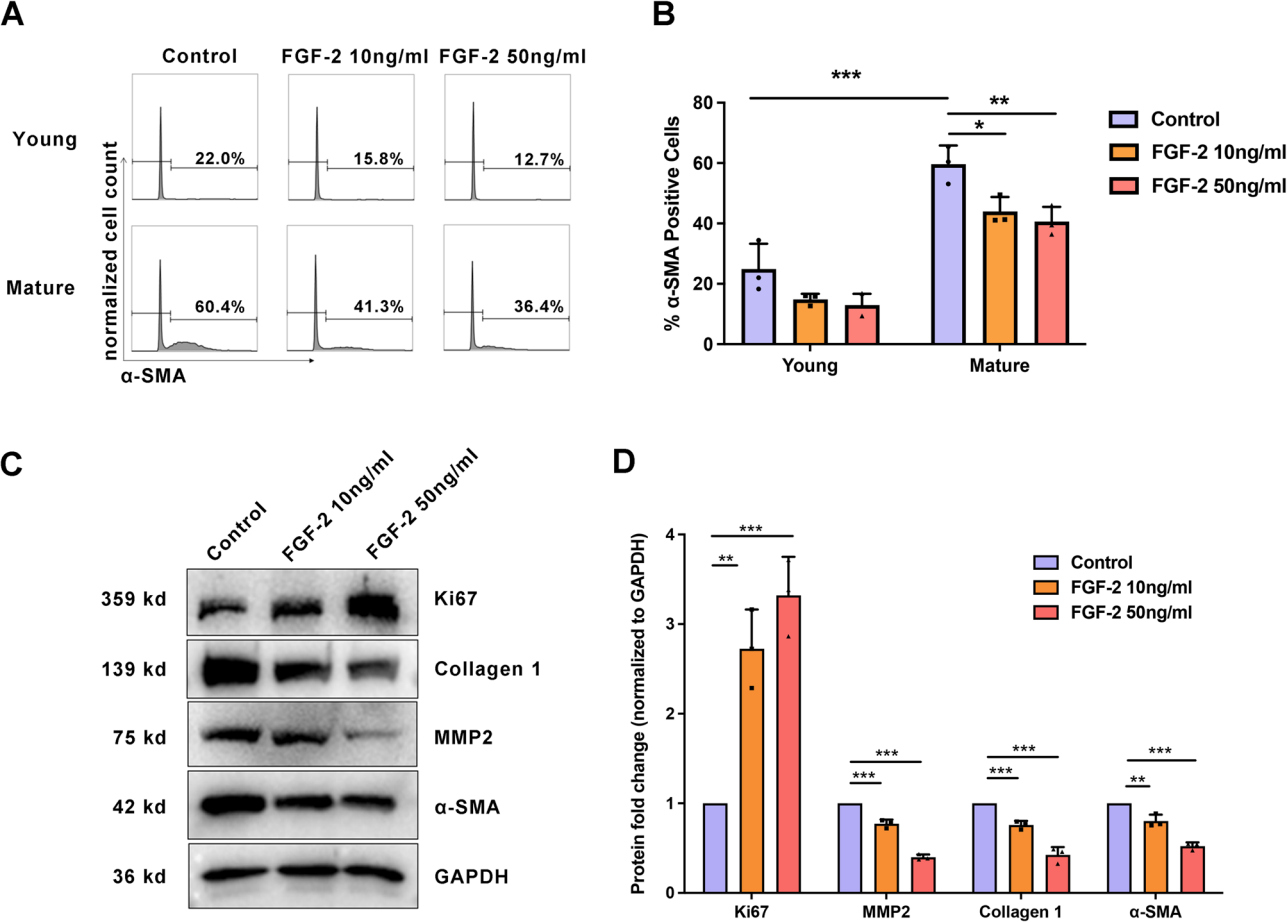
FGF-2 reversed age-related fibroblast-to-myofibroblast differentiation in scleral fibroblasts (SFs). Mature SFs were treated either with or without FGF-2 for 24 h. (**A, B**) Results represent a count of α-SMA positive cells using flow cytometry. ***P < 0.001, **P < 0.01, *P < 0.05, n = 3. (**C**) Western blot analysis was performed to probe for Ki67 (359 kDa), collagen 1 (139 kDa), MMP2 (75 kDa), and α-SMA (42 kDa). GAPDH was used as an internal control. (**D**) Quantification of the protein expression levels in **C**. The fold change relative to the control group level is displayed. ***P < 0.001, **P < 0.01, *P < 0.05, NS: not significant, n = 3.

Then, the SFs were treated with different concentrations of FGF-2. We found that mature SFs treated with FGF-2 showed more Ki67-positive cells than the control group (Fig. 4B and Fig. 5C, D), indicating promoted cell proliferation. Consistently, 10 ng/ml and 50 ng/ml FGF-2 promoted cell proliferation (0.46 ± 0.035 *versus* 0.65 ± 0.025 *versus* 0.84 ± 0.011 at 24 h, *P* < 0.001; Fig. 4A). Western blot and flow cytometry showed that the expression of α-SMA, MMP2, and collagen 1 was decreased after FGF-2 treatment, indicating that FGF-2 inhibits myofibroblast transition and changes ECM (Fig. 5A–D). For young SFs, the percentages of α-SMA positive cells were 24.9 ± 8.43 in the control group, 14.83 ± 1.85 in the 10 ng/ml FGF-2-treated group, and 12.96 ± 3.72 in the 50 ng/ml FGF-2-treated group. For mature SFs, the percentages of α-SMA positive cells were 59.63 ± 6.19 in the control group, 43.97 ± 4.79 in the 10 ng/ml FGF-2-treated group, and 40.63 ± 4.90 in the 50 ng/ml FGF-2-treated group (Fig. 5B).

## Discussion

Fibroblasts are cells that are inherent to the sclera, supporting ocular function and integrity. Around 25% of scleral thickness is occupied by fibroblasts^19^. SFs contribute to scleral remodeling in myopia^3^, however, the role of SFs during scleral tissue maturation is not yet well understood. In this study, we first demonstrated that SFs from posterior scleral shells are affected by natural maturation, becoming stiffer and exhibiting fibroblast-to-myofibroblast differentiation during maturation. *Fgf-2* and *Fgfrs* were upregulated during maturation as well. Exogenous FGF-2 increased cell proliferation and reversed the age-related fibroblast-to-myofibroblast differentiation.

The growth of axial length and the process of achieving emmetropia in guinea pigs exhibit similarities to humans^20^. In humans, axial length increases by 25% during the initial period of 2–3 years post-birth, while only by 1% over the subsequent ten years. In the guinea pig, axial length also increases more during first 3 weeks (12% infancy, 8% juvenile, 2% adolescent)^16,21^. Notably, myopia often occurs during juvenile stage. Therefore, in the present study, juvenile and adult guinea pigs have been selected as experimental subjects to investigate the process of scleral remodeling during emmetropization.

The sclera is an elastic, fibrous tissue, and there are race- and age-related differences in scleral elasticity and stiffness. Compared to the eyes of Europeans, for example, the eyes of Africans have higher shear stiffness and lower amounts of stretch^22^. Measuring the tangent modulus-stress–strain of the sclera demonstrated that the sclera becomes stiffer with aging^11^. In addition, there were regional variations in the biomechanical properties of the sclera. From the posterior to anterior regions of the sclera, extensibility decreased, and the tangent modulus increased^11,23^. However, all these studies used scleral tissue to analyze the biomechanical behavior of the sclera. There is little data on the change of SFs that might have an impact on sclera tissue. Our study appears to be the first to use AFM in the characterization of the biomechanical properties of living SFs in both young and mature guinea pigs and from the posterior to anterior regions of the sclera. We consistently found that SFs from posterior shells were stiffer than SFs from the equator in mature guinea pigs. Meanwhile, the SFs from the posterior shell became stiffer during maturation, while the SFs from the equator did not change.

Protein profile, proteoglycan, and ECM composition change during normal scleral growth^3,10,12^. Collagen makes up 50% of the scleral tissue by weight, with 95% of that collagen being type 1 (namely collagen 1)^24^. Evidence indicates that decreased expression of collagen 1 is responsible for ocular elongation and the thinning of the sclera^25^. Consistent with previous research^26^, scleral collagen 1 synthesis was higher than decomposition during the processes of maturation or emmetropization, since expression of collagen 1 increased in the SFs of mature guinea pigs. The main role of MMP2 is to degrade collagen 1 in the sclera, which can help maintain balance in scleral remodeling^27^. Our study found that the expression of MMP2 was increased, which was also consistent with previous research^26^. We speculated that the expression level of collagen 1 might play a pivotal role in maintaining scleral remodeling equilibrium, wherein reduced collagen 1 expression levels could serve as a molecular mechanism driving the transition from emmetropia to myopia.

Myofibroblasts are enriched with α-SMA, which augments their ability to generate contractile force and increases tissue stiffness^9^. Our results also showed that both the expression of α-SMA and the Young's modulus were increased. Like the changes in SFs related to myopia^8^, SFs also exhibited fibroblast-to-myofibroblast differentiation during maturation, which is characterized by a higher expression of α-SMA. The scleral stiffness increased in a form-deprivation myopia model using tree shrews as well^28^. Although further experiments are needed, we speculate that myopia might be a process of excessive emmetropia. This could be explained by ocular elongation, which is a common feature of both emmetropization and myopia.

FGF-2 is an established growth factor for cell proliferation, ECM remodeling, and inhibition of myopia^18,29,30^. Expression of scleral *Fgfr1* was significantly upregulated while endogenous *Fgf-2* was not significantly upregulated in myopic eyes^18^. Interestingly, our results first found that both *Fgf-2* and *Fgfrs* were significantly upregulated during maturation. We speculate that the upregulation of endogenous *Fgf-2* might help keep the eyes from excessive ocular elongation. Intravitreal application of FGF-2 has been shown to inhibit ocular elongation in the form of myopia deprivation (FDM) in a model using chicks^31^. In an FDM model using guinea pigs, peribulbar injection of FGF-2 also inhibited ocular elongation through upregulating collagen 1, integrin α2, and β1^14^. Our study consistently found that FGF-2 promoted cell proliferation. The FGF-2 regulated ECM by downregulating the expression of α-SMA, MMP2, and collagen 1, indicating inhibition of myofibroblast transition. The expression of collagen 1 resulted in a different type of change than that shown in a previous study, however, considering the different experimental models at play, more experiments are needed to understand this difference. This evidence as a whole indicates that FGF-2 is an effective mediator of controlling scleral remodeling and ocular elongation.

However, we acknowledge the limitations of our research, as we only included guinea pigs that were 4 weeks old and 6 months old in our study. To gain a comprehensive understanding of the changes in the sclera with age and regions, we would consider including guinea pigs of younger and older age groups and SF from different regions of sclera in future studies. Moreover, while we have evaluated the expression levels of *Fgf-2* and *Fgfrs* in SFs at the RNA level, it would be beneficial to investigate the change in protein levels in subsequent studies.

## Conclusion

In conclusion, our study first found that the fibroblast-to-myofibroblast differentiation was a normal part of scleral remodeling during maturation, with SFs from posterior scleral shells becoming stiffer. Both *Fgf-2* and *Fgfrs* were upregulated in mature SFs. Exogenous FGF-2 increased cell proliferation and reversed the age-related fibroblast-to-myofibroblast differentiation indicating that FGF-2 is an important mediator of scleral remodeling and ocular elongation. Although further translational studies are required, our study investigated the important roles of scleral fibroblast-to-myofibroblast differentiation and FGF-2 in the control of scleral remodeling during maturation.

## Materials and methods

### Animals

Healthy guinea pigs were purchased from Jiagan Biotechnology Co. Ltd (Shanghai, China). Pigmented guinea pigs were divided into two groups: young guinea pigs (four weeks old, randomly female and male) and adult guinea pigs (six months old, randomly female and male). They were reared in an animal facility under a 12-h light/dark cycle. Each experiment was repeated at least three times using different guinea pigs. At least 15 young and 15 adult pigmented guinea pigs were used in this study. We used an equal number of males and females in each experiment. Experiments were performed according to the ARVO Statement for the Use of Animals in Ophthalmic and Vision Research. This study protocol (No. 2014-004) was approved by the ethics committee of Zhongshan Ophthalmic Center, Sun-Yet Sen University. And this study is reported in accordance with ARRIVE guidelines. Refractive error and axial length measurements were obtained using streak retinoscopy and A-scan ultrasound (Supplementary Table 1).

### Cell isolation and culture

**Figure 6:**
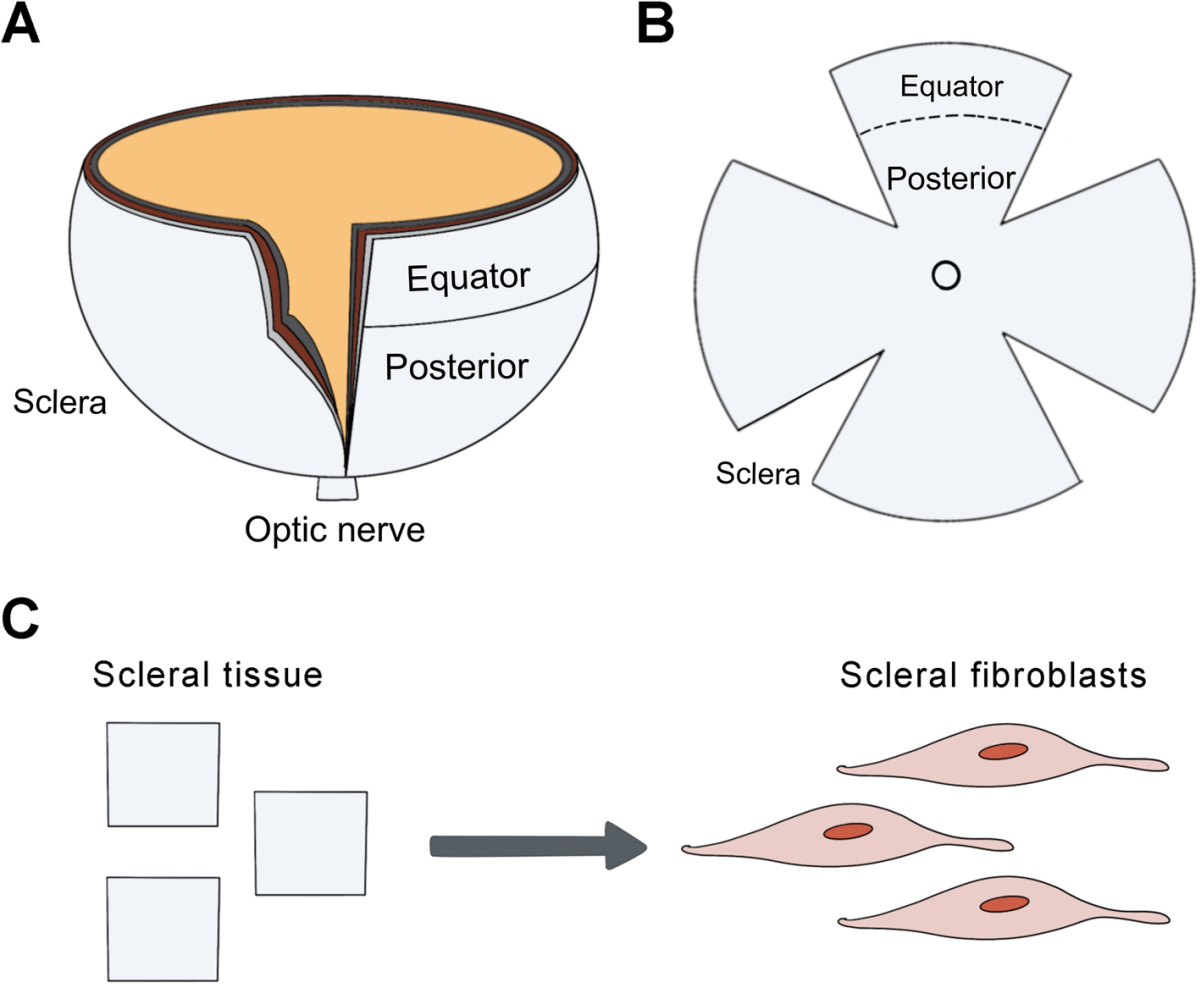
Cartoon schematic of equatorial and posterior sclera. The eyes of guinea pigs were harvested after euthanasia. (**A**) A circumferential incision was made along the limbus of the eyeball, then the anterior segment of the eye and the lens were discarded. (**B**) Then, the vitreous body, retina, and RPE-Bruch’s membrane-choroid complex were removed under a dissecting microscope. The scleral shells were divided into two regions: equatorial and posterior sclera. (**C**) The explanted tissues were cut and then placed in dishes for cell culture.

Guinea pigs were euthanized by overdose with an intraperitoneal injection of sodium pentobarbital and their right eyes were taken for further study. A circumferential incision was made along the limbus of the eyeball, the anterior segment of the eye and the lens were discarded, then the vitreous body, retina, and RPE-Bruch’s membrane-choroid complex were removed. Afterward, the scleral shells were divided into two regions, the equatorial and posterior sclera (Fig. 6A, B)^32,33^. The explanted tissues were cut into approximately 1 × 1 mm pieces (Fig. 6C), and then placed in 60 mm Nunc™ EasYDish™ (Thermo Fisher Scientific, Waltham, MA, USA) in Dulbecco’s modified Eagle’s medium (Nutrient Mixture F-12 [DMEM/F12; GIBCO, Grand Island, NY, USA]). Each 60 mm culture dish contained tissues from one animal. The medium was supplemented with a 10% HyClone™ Fetal Bovine Serum (FBS; GE Healthcare Life Sciences Company, Pittsburgh, PA, USA). The growth medium was replaced every 2–3 days. After 5–7 days of incubation at 37 °C and 5% CO_2_ in a humidified incubator, cells grew out of the scleral explants. Confirmation of the phenotype of primary cells was obtained by fluorescent immunostaining with vimentin, as described previously^34^.

### Atomic force microscopy (AFM)

A Dimension Fastscan with ScanAsyst™ AFM (Bruker, Santa Barbara, CA, USA) was used to analyze the biomechanical differences between young and mature groups of living SFs. The posterior and equatorial scleral shells of the guinea pigs were collected. After cell isolation, SFs were used to take atomic force microscopy measurements. The data were collected in at least six regions of each culture. An area of cells measuring 20 × 20 μm^2^ was imaged at a resolution of 128 × 128 pixels in contact mode, with a pyramidal tip of silicon nitride cantilevers (nominal spring constant k = 0.03N/m, MLCT-D, Bruker) selected. This test was performed as previously described^35^. A NanoScope Analysis (Bruker) was used to calculate cell elasticity values. The Young’s modulus was calculated using the Hertz model, which was selected from eight loci (500 × 500 nm) on each cell. The test temperature was controlled at 37 °C prior to AFM force indentation.

### Immunofluorescence

SFs were placed on MilliporeSigma™ Millicell™ EZ Slides (Thermo Fisher Scientific). Cells were fixed with 4% paraformaldehyde for 1 h, permeabilized with 0.1% Triton X-100 for 30 min, blocked by incubation in 1% bovine serum albumin in PBS (blocking solution) for 60 min. Cells were then incubated with one of the following: anti-vimentin-antibody (1:100; ab8978; Abcam, Cambridge, MA, USA), anti-α-SMA-antibody (1:100; ab5694; Abcam) or anti-Ki67-antibody (1:100; ab15580; Abcam) at 4 °C overnight, as well as with FITC-conjugated antibody (1:500; #4409 and #4412; Cell Signaling Technology, Danvers, MA, USA) at room temperature (RT) for 60 min. Lastly, the cells were incubated with 4′6-diamidino-2-phenylindole (DAPI) (1:1000 in PBS) at RT for 5 min and rinsed three times with PBS. Cells were imaged using a Zeiss LSM 510 confocal microscope.

### Cell proliferation assay

Cell counting kit-8 (CCK-8; Dojindo, Shanghai, China) was used to assess cell proliferation. The cells were cultured at a density of 1 × 10^4^ cells/well in a 96-well plate with a serum-free medium for 24 h. Cells were then treated with or without 10–50 ng/ml fibroblast growth factor 2 (FGF-2; Peprotech, Rocky Hill, NJ, USA) for another 24 h, 48 h and 72 h. Then, 10 μl CCK-8 solution was added and incubated at 37 °C for 2 h. The optical density (OD) values were detected using a 96-well multiscanner autoreader (Synergy H1, BioTek, Winooski, VT, USA) at 450 nm.

### Flow cytometry

Following a three-day-culture, SFs were digested with trypsin for 1 min and harvested. The cells were fixed and permeabilized with Fix/Perm solution (BD Bioscience, NJ, USA) for 15 min at 4 °C. Then the cells were incubated with anti-α-SMA-antibody (1:100; ab184675; Abcam) for 45 min in perm/wash buffer (BD Bioscience). All samples were acquired using BD LSR II (BD Bioscience) and data were analyzed with FlowJo v10 (Tree Star Inc, OR, USA).

### Western blot

SFs were scraped and lysed in ice-cold RIPA buffer (Beyotime Biotechnology, Shanghai, China), supplied with protease inhibitor (A32963, Thermo Fisher Scientific). Protein concentrations were determined using the BCA-100 Protein Quantitative Analysis Kit (Biocolor Bioscience & Technology Co., Ltd., Shanghai, China). Equivalent protein amounts (15 μg/lane) were separated on 10% SDS-polyacrylamide gels and electro transferred to Immobilon-P polyvinylidene fluoride membranes (Millipore Corporation, Bedford, MA, USA) using wet blotting. Then the PVDF membranes were blocked in 5% nonfat milk for 1 h. Blots were incubated with specific primary antibodies at 4 °C overnight and washed with PBST (0.1% Tween-20 in phosphate buffered saline). Then the blots were incubated with horseradish peroxidase- (HRP-)conjugated secondary antibodies (1:1000; #7074 and #7076; Cell Signaling Technology) at room temperature for 1 h. All the antibodies were diluted in PBST with 5% nonfat milk. After washed with PBST, signals were detected using the eECL Western Blot Kit (ComWin Biotech Co., Ltd, Beijing, China). Primary antibodies against α-SMA (1:250; ab5694; Abcam), collagen 1 (1:1000; ab88147; Abcam), Ki67 (1:1000; AF1738; Beyotime Biotechnology), matrix metalloproteinase2 (MMP2) (1:1000; AVARP20016_T100; Aviva Systems Biology, Beijing, China), and GAPDH (1:1000; 10494-1-AP; Proteintech, Rosemont, IL, USA) were used. Image J 1.48 (National Institutes of Health, Bethesda, MD, USA) was used to quantify the protein's signal grey density.

### Total RNA extraction and quantitative real-time PCR

Total RNA was extracted using TRIzol reagent and then transcribed into cDNA using a Transcriptor First Strand cDNA synthesis kit (Roche, Mannheim, Germany). To amplify and detect the target genes, a LightCycler 480 SYBR Green I Master (Roche) and a LightCycler480II real-time PCR system (Roche) were used. The LightCycler 480 SYBR Green I Master (Roche) was used to amplify the target genes, which were detected by using a LightCycler480II real-time PCR system (Roche). The relative mRNA levels were calculated using the $\Delta \Delta C_{t}$ method. C_t_ values > 40 were considered low efficiency and would not reported^36^. *GAPDH* were used as internal control. Primer sequences were as follows: *Fgf-2* (F), 5′-AGACTTCTCCGATGCTCCCA-3′; *Fgf-2*, (R), 5′-TAGCAAGGTAACGGTTCGCA-3′; *Fgf receptor 1 (Fgfr1)* (F), 5′-GAGGTGCTGACCCTGTTCAA-3′; *Fgfr1*, (R), 5′-AGGTAGAAGGGCGAGGTCAT-3′; *Fgfr2* (F), 5′-TGGTTGTGATCACCATGGCA-3′; *Fgfr2*, (R), 5′-CTTTCAACAGGCAGCGCAAT-3′; *Fgfr3* (F), 5′-CCATGGAGCGGCATGGACA-3′; *Fgfr3*, (R), 5′-TATCTGTCACACGCACACCG-3′; *Fgfr4* (F), 5′-AGAGGCTCCTCAAATGGGCA-3′; *Fgfr4*, (R), 5′-CAGCTGGACAGCGGAACTTA-3′; *GAPDH* (F), 5′-CCTTCCGTGTACCCACACC-3′; *GAPDH*, (R), 5′-AAGATGCCTTTGAGGGAGCC-3′.

### Statistical analysis

Statistical analyses were performed using SPSS 19.0 (SPSS, Chicago, IL, USA). Statistical significance was determined with an analysis of variance (ANOVA), followed by Tukey’s and Bonferroni post-hoc analysis. The Bonferroni post-hoc test was used when variances were equal, while Tukey’s post-hoc test was used when they were not. Because the data were not normally distributed, a nonparametric test (two-sided Wilcoxon rank-sum test) was performed on the AFM measurements. All values were presented as mean ± SD. Results were considered statistically significant if values were *P* < 0.05.

### Supplementary Information

Supplementary Information 1.

Supplementary Information 2.

Supplementary Information 3.

## Data Availability

All data relevant to this study is available upon reasonable request from the corresponding author.
