# Supplementary material for: Scleral remodeling in early adulthood: the role of FGF-2
Source: Sci Rep. 2023 Nov 27;13:20779. doi: 10.1038/s41598-023-48264-5 (PMC10682392; doi:10.1038/s41598-023-48264-5)
Supplement: Supplementary file 1 — Supplementary Information 1. [file 41598_2023_48264_MOESM1_ESM.docx]

Amplification curves for *Fgf-2*


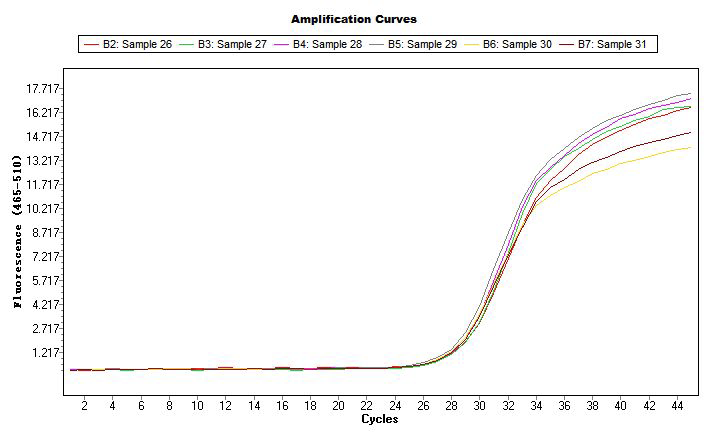


Amplification curves for *Fgfr1*

**
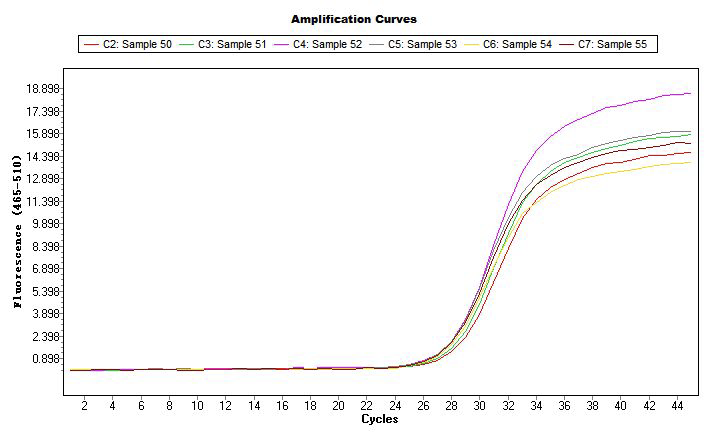
**

Amplification curves for *Fgfr2*

**
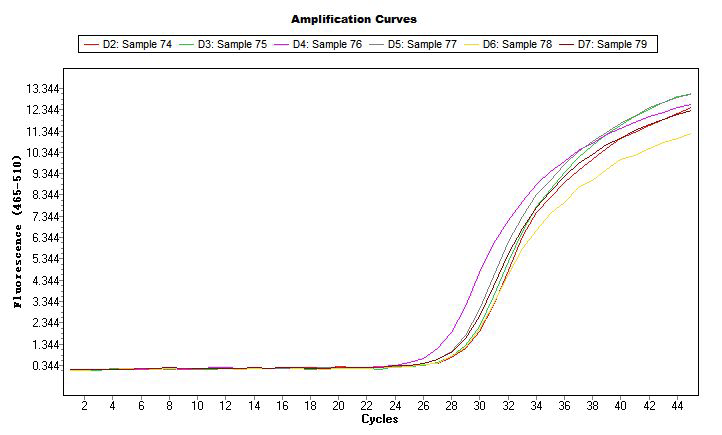
**

Amplification curves for *Fgfr3*

**
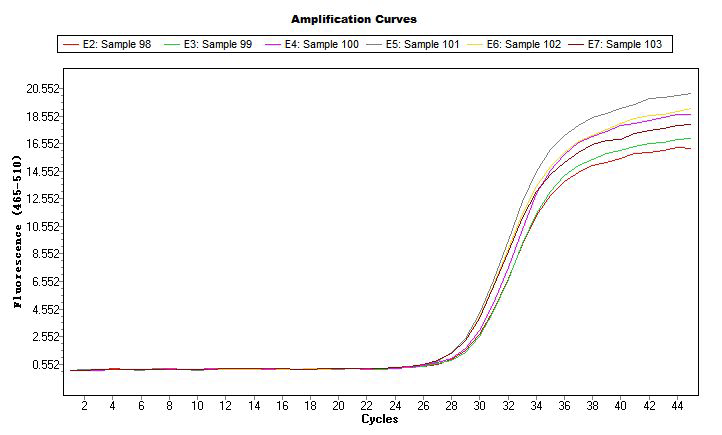
**

Amplification curves for *Fgfr4*

**
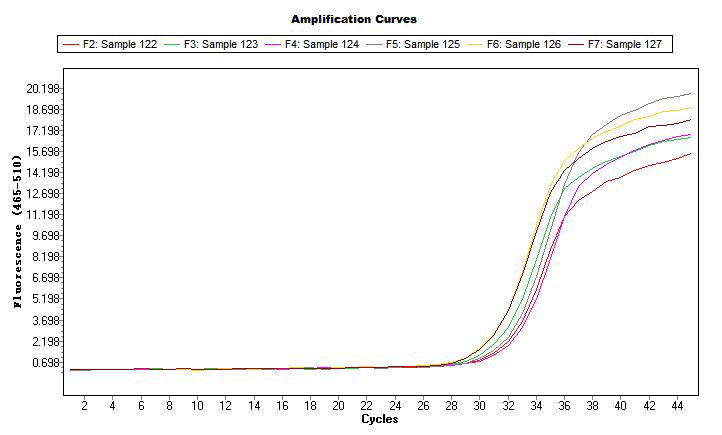
**

Amplification curves for *GAPDH*

**
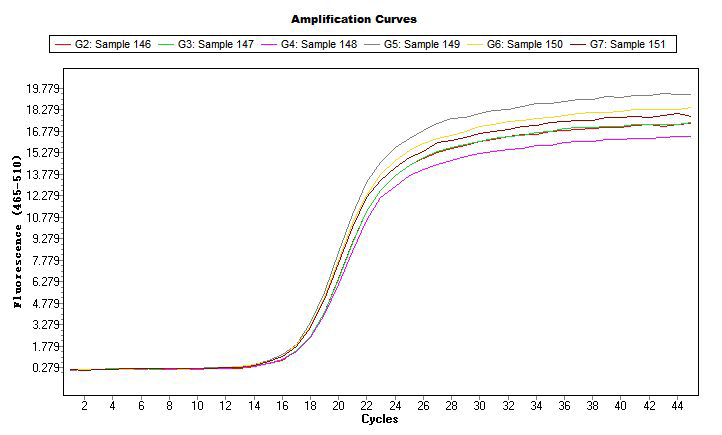
**
