# Supplementary material for: Scleral remodeling in early adulthood: the role of FGF-2
Source: Sci Rep. 2023 Nov 27;13:20779. doi: 10.1038/s41598-023-48264-5 (PMC10682392; doi:10.1038/s41598-023-48264-5)

Full-length blots of three experiments were provided.

GAPDH for Figure 2B


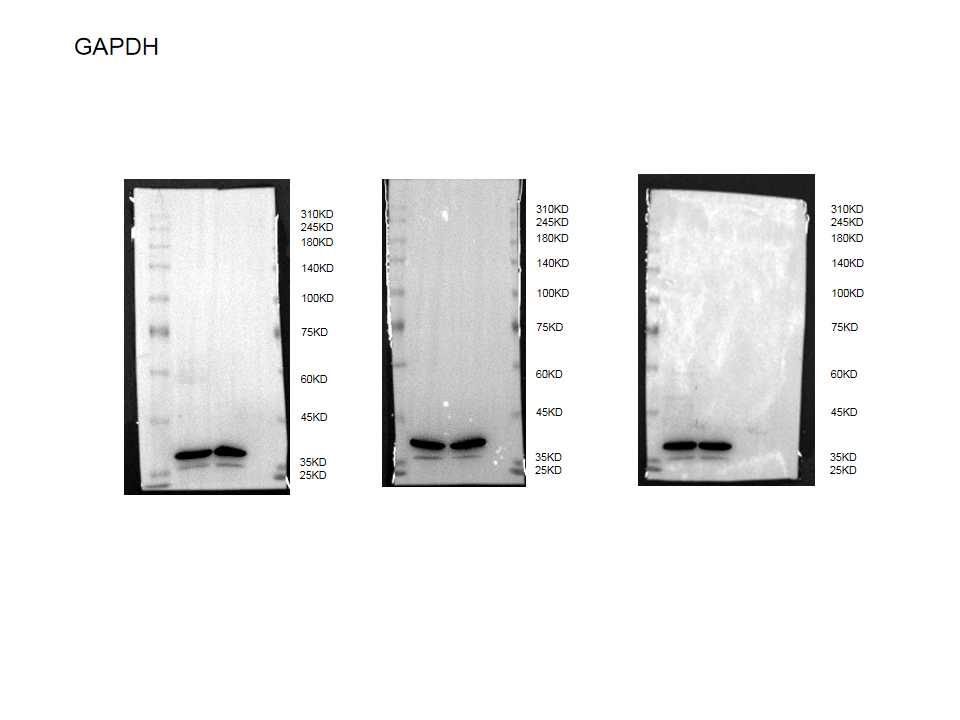


α-SMA for Figure 2B


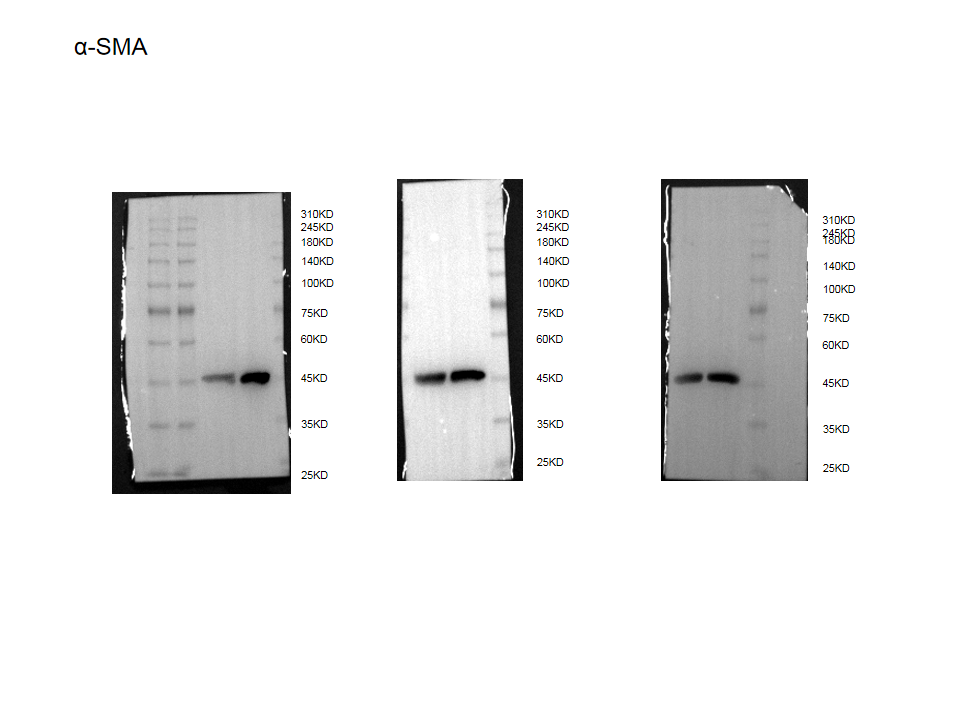


MMP2 for Figure 2B


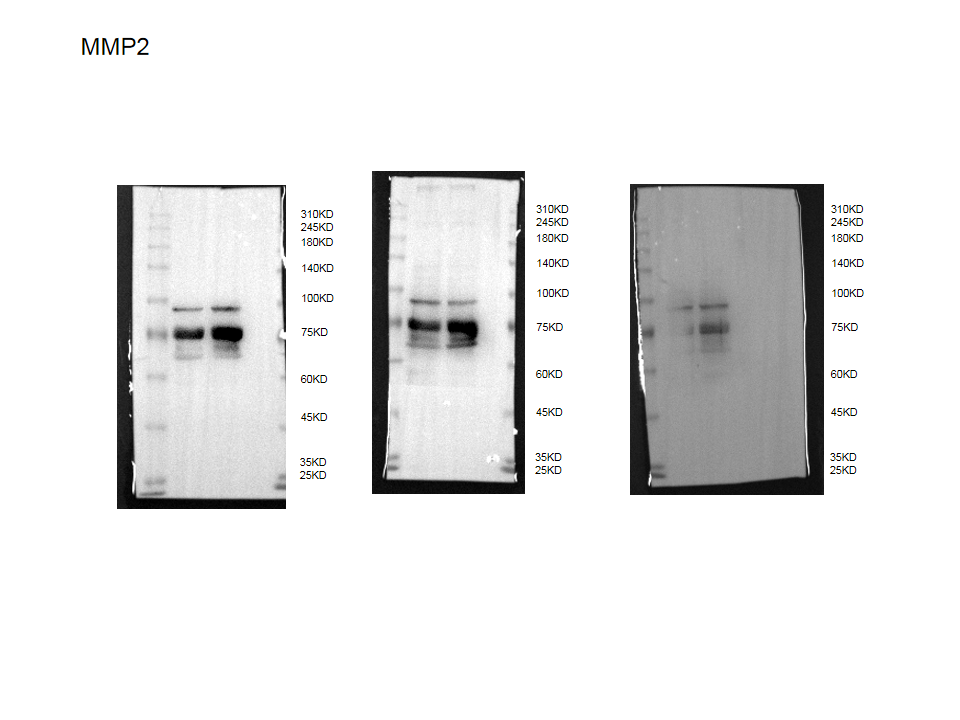


Collagen 1 for Figure 2B


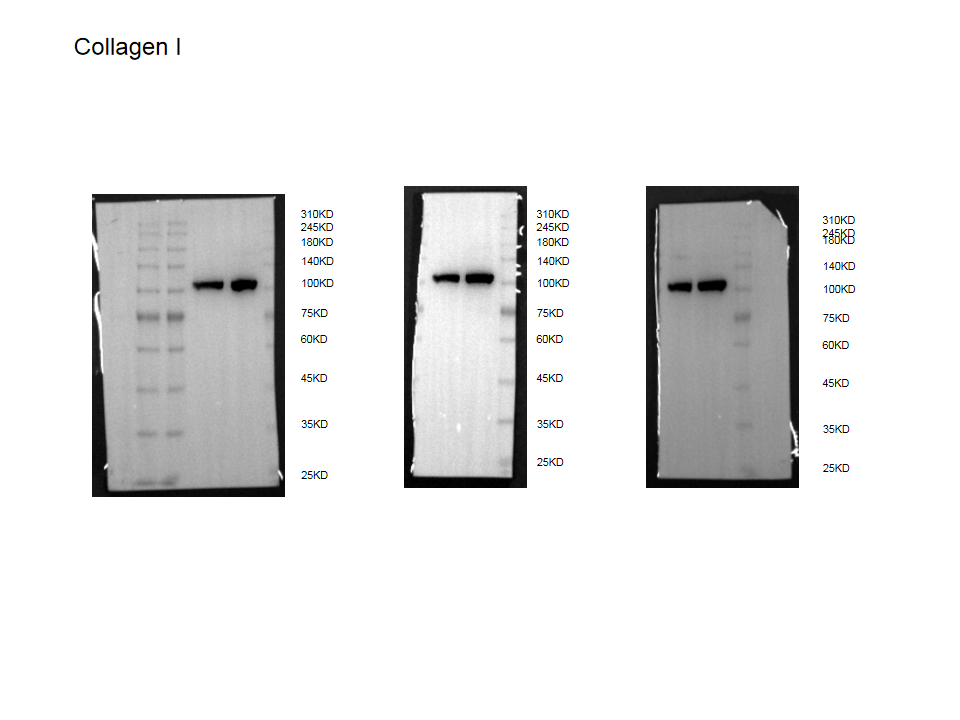


Ki67 for Figure 2B


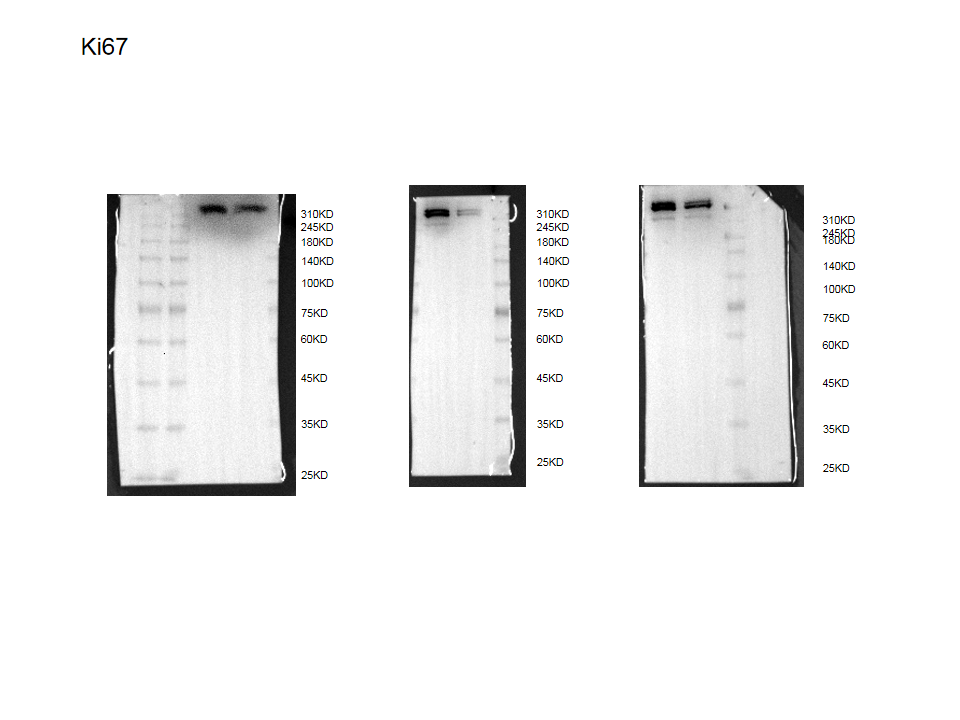


GAPDH for Figure 5C


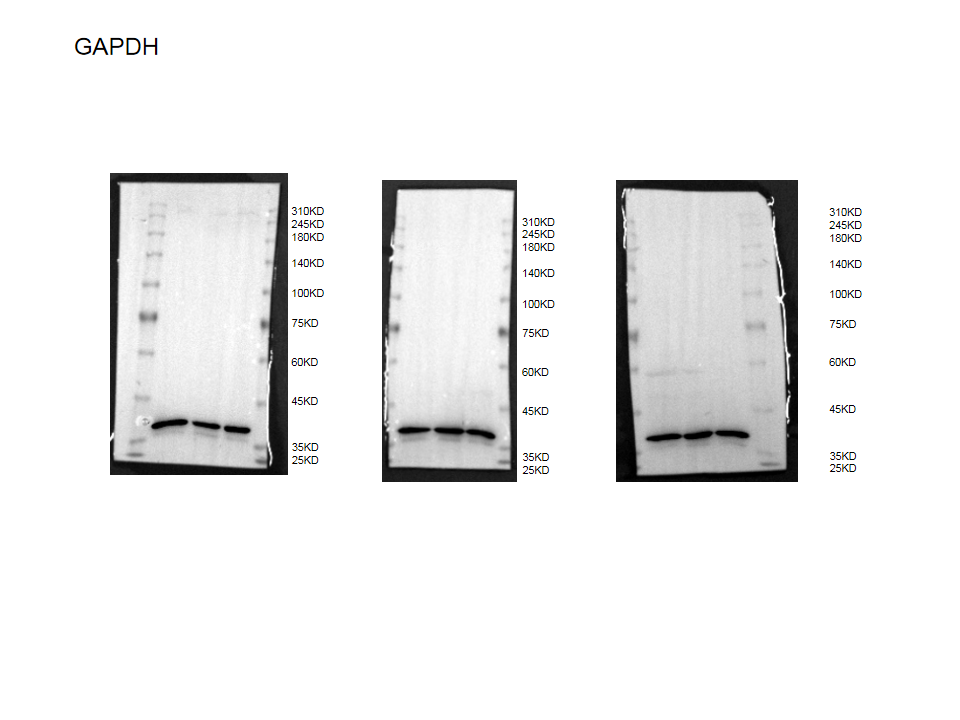


α-SMA for Figure 5C


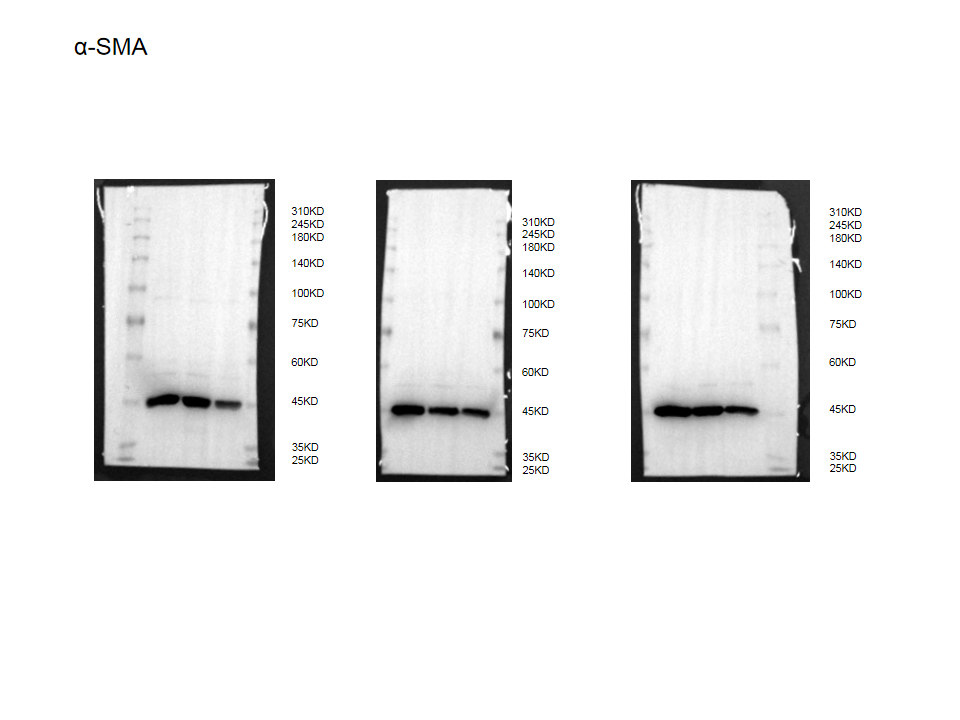


MMP2 for Figure 5C


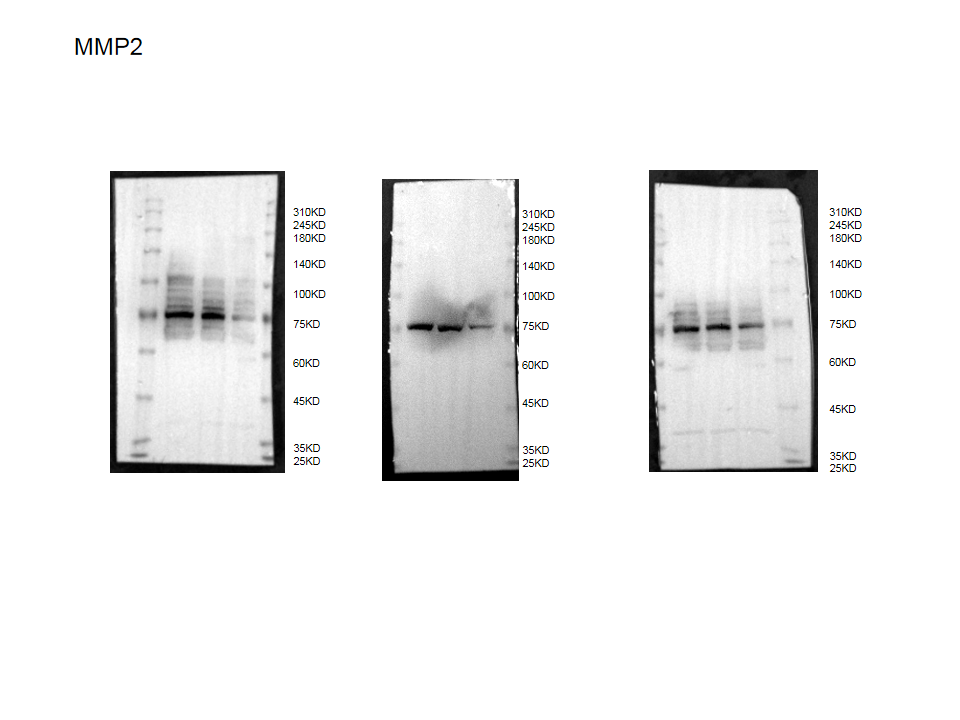


Collagen 1 for Figure 5C


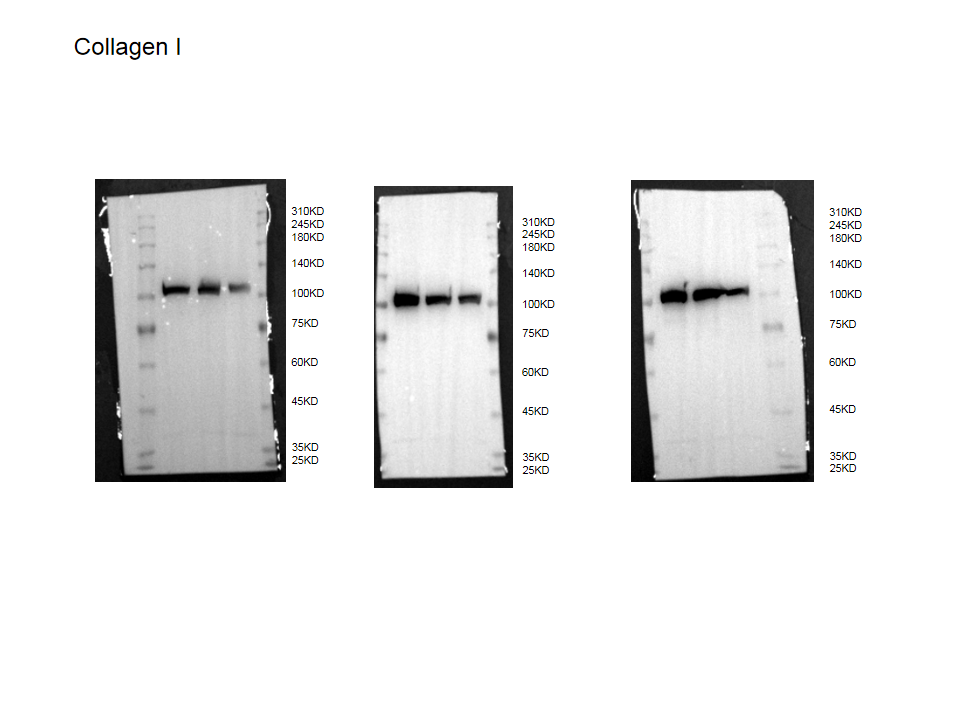


Ki67 for Figure 5C


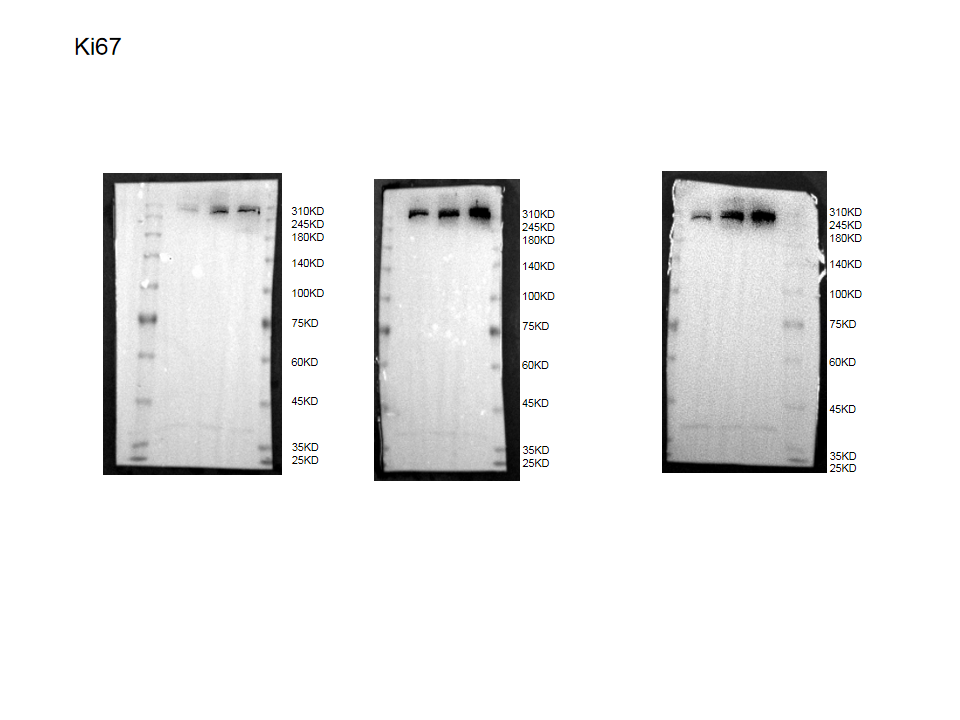

Supplement: Supplementary file 2 — Supplementary Information 2. [file 41598_2023_48264_MOESM2_ESM.docx]
