# Supplementary material for: Scleral remodeling in early adulthood: the role of FGF-2
Source: Sci Rep. 2023 Nov 27;13:20779. doi: 10.1038/s41598-023-48264-5 (PMC10682392; doi:10.1038/s41598-023-48264-5)
Supplement: Supplementary file 3 — Supplementary Information 3. [file 41598_2023_48264_MOESM3_ESM.docx]

Supplementary Materials

**Supplementary Table 1. Ocular parameters for young and mature guinea pigs.**

|  | 4 weeks, Mean ± SD | 6 months, Mean ± SD |
| --- | --- | --- |
| Refractive error, D | 4.54 ± 0.43 | 2.08 ± 0.41 |
| Axial length, mm | 8.12 ± 0.25 | 9.33 ± 0.14 |
